# Income and immunity: The consequences of a pre- and neo-natal income shock on childhood infection risk

**DOI:** 10.23889/ijpds.v8i2.2190

**Published:** 2023-09-14

**Authors:** Mary-Alice Doyle, Stefanie Schurer

**Affiliations:** 1 London School of Economics, London, United Kingdom; 2 University of Sydney, Sydney, Australia

## Objectives

The experiences and conditions that children are exposed to in utero and early infancy impact their health in adulthood. Understanding the channels through which these effects emerge is important, because this may help us to anticipate which children are at highest risk of adverse effects, and provide early interventions to help. In this paper, we test a new channel linking perinatal experiences to later-life health: immunity.

## Method

We use administrative birth records, linked with hospital admissions episodes and primary care consultations for children in the Northern Territory of Australia. This data forms part of the Child and Youth Development Partnership data linkage project. We analyse the impact of a shock to household income in Aboriginal communities, resulting from a change in government transfer policy. We estimate the impact of exposure to this shock in utero or in the first 3 months of life, relative to exposure later in childhood, to isolate the impact of an income shock during this key developmental period.

## Results

 We find that children who were exposed to the shock in utero or in their first three months of life were at higher risk of severe infection requiring hospital admission. They spend, on average, 4.5 more days in hospital from birth to their 6th birthday (a 45 percent increase). Most of this impact is concentrated in admissions for infection. Only a small share of the effect can be explained by the immediate impact of the policy on birthweight.

## Conclusion

Our findings are consistent with the explanation that an income shock worsens nutrition, and that worse nutrition during key developmental stages can permanently weaken children’s immune systems. This finding speaks to the importance of attention to key phases in childhood development, when designing policies that affect households’ financial resources. Furthermore, we find birthweight is a poor predictor of childhood hospital admission. However, use of longitudinal administrative data helps us to identify alternative markers of new-born health that are better predictors.

